# Antigenicity and immunogenicity of a novel, acute HIV-1 Tanzanian subtype C gp145 envelope protein for clinical development

**DOI:** 10.1186/1742-4690-9-S2-P323

**Published:** 2012-09-13

**Authors:** V Polonis, L Wieczorek, V Kalyanaraman, G Matyas, S Whitney, C Williams, S Tovanabutra, E Sanders-Buell, M Wesberry, C Ochsenbauer, A Chenine, M Rao, T Tong, C Alving, H Cheng, S Zolla-Pazner, N Michael, T VanCott, M Marovich

**Affiliations:** 1 Walter Reed Institute of Research, DIV Retrovirology, Silver Spring, MD, USA; 2 Henry M. Jackson Foundation, Silver Spring, MD, USA; 3 Advanced Biosciences Laboratories, Inc, Rockville, MD, USA; 4 New York University School of Medicine, New York, NY, USA; 5 University of Alabama, Birmingham, AL, USA; 6 University of California, Davis, CA, USA

## Background

Eliciting broadly reactive neutralizing antibodies remains a challenge in HIV-1 vaccine development, complicated by variations in envelope (Env) subtype and structure, and by the assays used for product down-selection. Since a majority of new HIV-1 infections are subtype C and considering the novel properties of C Envs, a C Env (CO6980v0c22) from an acutely infected Tanzanian was developed as a candidate HIV vaccine.

## Methods

The CO6980v0c22 Env sequence was codon optimized and a stable CHO cell line expressing gp145 was established. Purified gp145 was adjuvanted in alum or lipid A-liposomes, injected into New Zealand white rabbits (4/group; 25 ug at weeks 0, 4, and 8), or BALB/c mice (5/group; 10 ug in liposomes at weeks 0, 3, 6, 8). Antibody titers were assessed by ELISA and neutralizing antibodies were measured against pseudoviruses in TZM-bl cells or against infectious molecular clones (IMC) in a PBMC assay.

## Results

Secreted gp145 is a novel subunit with the full MPER extended by three lysines. Unlike some gp140 subunits, the 4E10 neutralizing monoclonal antibody (mAb) binds to gp145. IgG1b12 binds weakly, VRC01 binds potently, as does the V2-specific 697D mAb; the gp145 also binds to alpha4beta7 receptor, as demonstrated by flow cytometry. At week 10 post-immunization, rabbit sera showed strong binding antibody titers to several Env antigens, including the clade B V1V2gp70 scaffold protein. While neutralization of the HIV-2 MPER-scaffold pseudovirus was negative, cross-clade neutralization was observed in both rabbits and mice, against Tier 1 subtype B and C pseudoviruses, and against Tier 1 and Tier 2 IMC. Using EGS cross-linking, it appears that the majority of the gp145 multimers are trimeric; this is currently under investigation using electron microscopy techniques.

## Conclusion

These data indicate essential immunogenic features of a novel acute C HIV-1 Env that warrants further testing for potential clinical development.

